# Inhibition of Breast Cancer Cell Proliferation and *In Vitro* Tumorigenesis by a New Red Apple Cultivar

**DOI:** 10.1371/journal.pone.0135840

**Published:** 2015-08-18

**Authors:** Giuditta Fiorella Schiavano, Mauro De Santi, Giorgio Brandi, Mirco Fanelli, Anahi Bucchini, Laura Giamperi, Giovanna Giomaro

**Affiliations:** 1 Department of Biomolecular Sciences, University of Urbino “*Carlo Bo*”, Urbino (PU), Italy; 2 Department of Earth, Life and Environmental Sciences, University of Urbino “*Carlo Bo*”, Urbino (PU), Italy; University of Kentucky, UNITED STATES

## Abstract

**Purpose:**

The aim of this study was to evaluate the antiproliferative activity in breast cancer cells and the inhibition of tumorigenesis in pre-neoplastic cells of a new apple cultivar with reddish pulp, called the Pelingo apple.

**Methods:**

The antiproliferative activity was evaluated in MCF-7 and MDA-MB-231 human breast cancer cells. The inhibition of tumorigenesis was performed in JB6 promotion-sensitive (P+) cells.

**Results:**

Results showed that Pelingo apple juice is characterized by a very high polyphenol content and strongly inhibited breast cancer cell proliferation. Its antiproliferative activity was found to be higher than the other five apple juices tested. Pelingo juice induced cell accumulation in the G2/M phase of the cell cycle and autophagy through overexpression of p21, inhibition of extracellular signal-regulated kinases 1/2 (ERK1/2) activity and an increase in lipidated microtubule-associated protein-1 light chain-3 beta (LC3B). Remarkably, Pelingo juice inhibited the 12-o-tetra-decanoyl-phorbol-13-acetate (TPA)-induced tumorigenesis of JB6 P+ cells, suppressing colony formation in semi-solid medium and TPA-induced ERK1/2 phosphorylation.

**Conclusions:**

Our data indicate that the Pelingo apple is rich in food components that can markedly inhibit *in vitro* tumorigenesis and growth of human breast cancer cells and could provide natural bioactive non-nutrient compounds, with potential chemopreventive activity.

## Introduction

Several epidemiologic studies suggest that diets rich in fruits and vegetables may reduce the risk or delay the development of chronic diseases such as cancer, cardiovascular disease and diabetes [1]. The idea that these natural foods might help to reduce the risk for various types of cancer, including breast cancer, dates back several decades [2]. It is estimated that about one third of all cancer deaths could be prevented by increasing consumption of fruits, vegetables, and whole grains [3–6].

Many of these protective effects have been attributed to non-nutrient plant constituents such as carotenoids, phenolic acids and flavonoids [1, 7–12]. Among fruits, apples contain an extraordinary selection of such bioactive phytochemicals [13]. Indeed, they have the second highest level of antioxidant activity and content of phenolic compounds of all fruits [14]. Apples are a very significant part of the human diet and a daily intake of apples has been associated with the prevention of several chronic diseases [15], including different types of cancers [8, 16, 17].

The anticancer activity of apple constituents has also been documented in rat models. Whole apple extract has been reported to prevent breast cancer in a dose dependent manner [6,17]. Moreover, in vivo studies have shown the cancer prevention potential based on the ability of the juice to reduce genotoxicity, hyperproliferation and the development of aberrant crypt foci experimentally induced in a rat model by dimethylhydrazine [18].

The antiproliferative properties of apple extracts have been described extensively by *in vitro* studies. Apple have reportedly shown potent antiproliferative activity against human liver cancer HepG2 cells, human colon cancer Caco-2 cells and estrogen receptor-positive (ER+) (MCF-7) and triple-negative (MDA-MB-231) human breast cancer cell lines [19–22]. Further, a flavonoid mixture from apples has been shown to inhibit the proliferation of HT29 cells [23]. Raw extracts from apple waste have been shown to protect against DNA damage and inhibit the invasion of colon cancer cells [24]. Non-extractable polyphenols from industrial apple waste have shown efficacy against the proliferation of several human cancers cells, such as human cervical (HeLa), human hepatoma (HepG2), and human colon cancer cells (HT-29) [25]. The protective effects of apples have been attributed primarily to their anti-oxidant properties. Conversely, it has been reported that phenolics with poor anti-oxidant properties are able to inhibit proliferation of CaCo-2 and HT29 cells and to increase apoptosis, suggesting independent anti-oxidant mechanisms [26]. However, the molecular mechanisms of the anticancer properties of apple phytochemical are not completely understood.

This study focuses on a recently identified apple, named Pelingo apple, characterized by reddish colour and sweet fruity flavour [27–29]. These characteristics distinguish it from other apple types with reddish pulp which are often not very tasty or sour and therefore unmarketable.

Moreover, this apple contains an appreciable amount of polyphenols also in the pulp that showed good antioxidant activity comparable to that of red berries [30].

This makes the discovery of this new cultivar with potential health benefits quite interesting, and its propagation has been entrusted to a consortium of the breeders.

The purpose of this investigation was to evaluate the antiproliferative activity in both MCF-7 and MDA-MB-231 human breast cancer cell lines and the inhibition of tumorigenesis in JB6 Cl 41-5a promotion-sensitive (JB6 P+) of the Pelingo apple juice. Our results show that Pelingo juice is characterized by a very high polyphenol content, strongly inhibits the proliferation of human breast cancer cells through accumulation in the G2/M phase of the cell cycle, overexpression of p21 and inhibition of extracellular signal-regulated kinases 1/2 (ERK1/2) activity. Moreover, Pelingo juice inhibited the 12-o-tetra-decanoyl-phorbol-13-acetate (TPA)-induced tumorigenesis of JB6 P+ cells.

## Materials and Methods

### Preparation of apple juice

We have screened six apple cultivars to assess their antiproliferative activity on breast cancer cell lines: the Pelingo apple (S1 Fig), which is an autochthonous red apple cultivar grown in hilly areas of the Pesaro-Urbino province, Central Italy, and the commercially available apples Abbondanza red pulp, Abbondanza white pulp, Marchigiana pink pulp, Annurca white pulp and Bacci white pulp. All the cultivars were harvested when fully ripened (August- September).

The apples were washed in tap water and dried. The apple core was removed and the remaining parts, cut into small pieces and homogenized using a household juicer (Moulinex JU200045). The solid matrix was then separated by a first centrifugation at 3000 rpm and juice was collected and centrifuged at 10000 rpm for 10 min at 4°C. Finally, the juice supernatant was filtered through a 0.45 μm membrane and stored in aliquots at -20°C.

### Determination of total apple juice polyphenols and anthocyanins

The total anthocyanin content of the apple extracts was measured using the differential pH method reported by Elisia et al. [31] and Tzulker et al. [32]. Two aliquots of juice (sample) were dissolved separately in potassium chloride buffer (KCl 0.025 M, pH 1.0) and sodium acetate (CH_3_CO_2_Na 3H_2_O, 0.4M, pH 4.5). The absorbance measurements of the samples were read at 510 and 700 nm against a control cell containing solvent instead of sample (in the same quantity). The absorbance (A) of the diluted sample was then calculated as follows:

$$\text{A= }{\left({\text{A}}_{\text{510nm}}{\text{-A}}_{\text{700nm}}\right)}_{\text{pH }1.0}\text{-}{\left({\text{A}}_{\text{510nm}}{\text{-A}}_{\text{700}}\right)}_{\text{pH }4.5}$$ (1)

The monomeric anthocyanin pigment concentration in the original sample was calculated as reported by Elisa et al. and Tzulker *et al*. [31, 32]. The total content of the polyphenolic compounds was determined by the Prussian Blue method [33] with slight modifications. Aliquots of the juice were made using up to 1 mL of distilled water. After adding 60 μL of 0.1 M FeNH_4_(SO_4_)_2_, they were incubated for 20 min at room temperature (22°C). Subsequently, 60 μL of 8 mM K_3_Fe(CN)_6_ were added to the sample, and after 20 min at room temperature the optical density of the mixture was determined at 720 nm (Jasco V-530 spectrophotometer, Tokyo, Japan). Quercetin (Sigma-Aldrich) was used as the standard to construct a calibration curve.

### Cell culture and reagents

MCF-7 and MDA-MB-231 cell lines, diploid human fibroblasts from fetal lung tissue (WI-38), embryo mouse fibroblasts (NIH-3T3) and murine skin epidermal (JB6 P+) cells were purchased from the American Type Cell Culture Collection. All cell lines (with exception of JB6 P+) were cultured in DMEM supplemented with 10% of heat-inactivated FBS, 2 mM glutamine, 0.1 g/L streptomycin, 100 units/ml penicillin and, only for MCF-7, 10 μg/ml insulin. JB6 P+ cells were cultured in EMEM supplemented with 5% of heat-inactivated foetal bovine serum (FBS), 2 mM glutamine, 0.1 g/L streptomycin, 100 units/ml penicillin and 1 mM Na-pyruvate. The cells were grown in a humidified atmosphere at 37°C with 5% CO_2_. TPA was dissolved in DMSO and used to a final concentration of 10 ng/ml. All cell culture materials were purchased from Sigma-Aldrich (St. Louis, MO, USA).

### Anchorage-dependent growth assay

MCF-7 and MDA-MB-231 cells were plated at a density of 5x10^4^ cells/well in 12-well plate and JB6 P+ cells were plated at a density of 3x10^5^ cells/well in 35 mm dishes, incubated at 37°C with 5% CO_2_ overnight and treated with increasing concentrations of Pelingo juice (only JB6 P+ were stimulated with 10 ng/ml TPA). After 72 h of treatment cells were washed in PBS and counted using a hemacytometer by Trypan blue exclusion assay or fixed in 4% formaldehyde for 15 min and stained with 0.1% crystal violet for 15 min. Pictures were captured using an optic microscope (20X).

### Anchorage-independent transformation assay (soft-agar assay)

Assays were carried out in 35 mm dishes. For each dish, the bottom layer consisted of 1 ml of 0.6% agar in DMEM or EMEM (10% FBS) for MDA-MB-231 and JB6 P+ respectively. A total of 5x10^3^ MDA or 5x10^4^ JB6 P+ cells suspended in 1 ml of 0.3% agar in complete medium, were layered on top. Both layers were supplemented with Pelingo juice and, only in JB6 P+, with TPA (10 ng/ml) or vehicle (DMSO 0.01 μl/ml). Weekly, 500 μl of complete medium was added to maintain humidity. Cells were incubated at 37°C for 14 days (MDA- MB-231) and for 21 days (JB6 P+). The colonies after crystal violet staining (0.01%) were counted and photographed with an inverted microscope (5X). Only clusters containing more than 20 cells were counted as colonies.

### Cell viability Assay

Cells were plated at a density of 5x10^3^ cells/well in 96-well plate, incubated at 37°C overnight and treated in triplicate with increasing concentrations of apple juice. After 72 h of treatment, cell viability was evaluated using the CellTiter 96 Aqueous Non-Radioactive Cell Proliferation Assay (Promega, Madison, WI, USA), based on the ability of viable cells to convert a soluble tetrazolium salt (3-(4,5-dimethylthiazol-2-yl)-5-(3-carboxymethoxyphenyl)-2-(4-sulfophenyl)-2H-tetrazolium, MTS) to a formazan product, as reported previously [34]. The results were expressed as relative viable cells compared to controls (untreated cells). The 50% inhibitory concentration (IC_50_) values of antiproliferative activity were calculated by nonlinear regression analysis using the equation:

$$y\text{= 1 - Top·}x^{\text{(HillSlope)}}\text{/}\left({\text{IC}}_{\text{50}}{}^{\text{(HillSlope)}}\text{+}x^{\text{(HillSlope)}}\right)$$ (2)

(Prism5; GraphPad Software, Inc., La Jolla,CA, USA).

### Proliferative index

MCF-7, MDA-MB-231, NIH-3T3 and WI-38 cells were seeded at a density of 1x10^5^ cells in 3 ml of appropriate growth medium in 30 mm dishes for 24, 48, 72 and 96 h. Proliferative indices were evaluated using the formula:

$$\left({\text{N}}_{\text{2}}{\text{-N}}_{\text{1}}{\text{/ T}}_{\text{2}}{\text{-T}}_{\text{1}}\right){\text{/N}}_{\text{1}}$$ (3)

where N represents the cellular count and T the time.

### Cell cycle analysis

Cell cycles were analyzed by means of the propidium iodide staining procedure previously reported [35]. Briefly, cells were washed twice with PBS, fixed with ice-cold 70% ethanol at 4°C for 1 h and stained with propidium iodide staining solution in PBS 1X (0.1% sodium citrate, 0.1% Triton X-100, 250 μg/mL RNase A, and 50 μg/mL propidium iodide). Cytofluorimetric acquisitions and sample analysis were performed with a Partec PAS flow cytometer (Partec, Münster, Germany) and FlowJo 8.6.3 software (TreeStar, Inc., Ashland, OR, USA), respectively.

### Apoptosis/necrosis/autophagy evaluation

Apoptosis, necrosis and autophagy induction was evaluated as previously described [34]. Briefly, MDA-MB-231 and MCF-7 cells were seeded at 1x10^5^ cells per 35 mm dish and allowed to attach overnight. Cells were treated with 2.5 and 5% v/v Pelingo juice for 24, 48 and 72 h. After treatments, cells were directly stained propidium iodide, Hoechst and acridine orange, viewed with a fluorescence microscope as reported previously [34]. Cells treated with Paclitaxel 1 μM for 24 h and H_2_O_2_ 3 mM for 1 h were used as positive controls for apoptosis and necrosis, respectively.

### Immunoblot analysis

MCF-7, MDA-MB-231 and JB6 P+ cells were seeded at 5x10^5^ cell in 30 mm diameter dishes, attached overnight and treated with Pelingo juice (only JB6 P+ were stimulated with 10 ng/ml TPA). At indicated times, cells were washed with ice-cold PBS and lysed as reported in [34]. Proteins extracted were fractionated on 12% (p21, microtubule-associated protein-1 light chain-3 beta, LC3B) and 7.5% (ERK1/2, phospho-ERK1/2) SDS-PAGE and then electrically transferred to Trans-Blot transfer medium (0.2 μm) nitrocellulose membrane (Bio-Rad Laboratories, Inc.). Blots were incubated with anti-p21 antibody purchased from Santa Cruz Biotechnology, Inc. (Santa Cruz, CA, USA), anti-ERK1/2, anti phospho-ERK1/2 and anti-LC3B antibodies purchased from Cell Signaling Technology (Danvers, MA, USA) overnight at 4°C and then incubated for 1 h at room temperature with peroxidase-conjugated secondary antibody. Blots were treated with enhanced chemiluminescence reagents, and all of the proteins were detected and quantitated by Chemi-Doc System (Bio-Rad Laboratories, Inc.). Equal protein loading was confirmed by the level of actin protein present in the membrane tested with anti-actin antibody (Sigma-Aldrich).

### Statistical analysis

The results are presented as means ± SEM of at least 3 separate experiments. Data were analyzed using 1- or 2-way ANOVA as appropriate followed by Bonferroni or Dunnet’s *post hoc* tests. Differences were considered significant at *p<*0.05. All statistical analyses were performed using Prism5 software.

## Results

### Quantification of apple juice polyphenols and anthocyanins

The amounts of polyphenols and anthocyanins measured in apple juice from the six cultivars are reported in Table 1. The data clearly show that total polyphenol content was higher in Pelingo juice (1.996 mg/ml) than it was in the other cultivars, whereas anthocyanins were more abundant in Abbondanza red pulp juice (136.7 μg/ml) than in Pelingo juice (28.39 μg/ml). As expected, the amounts of anthocyanins measured in the cultivars of white pulp were much smaller.

**Table 1:** IC_50_ values (% v/v of fresh juice of all varieties of apple) obtained in MCF-7 and MDA-MB-231 and polyphenol and anthocyanin content of apple juices.

	MCF-7	MDA-MB-231	Polyphenols (mg/ml)	Anthocyanins (μg/ml)
Abbondanza white pulp	nd	5.93 ± 0.21	0.294	6.70
Abbondanza red pulp	8.15 ± 0.46	3.51 ± 0.19	0.558	186.69
Marchigiana pink pulp	nd	12.24 ± 0.71	0.219	0.83
Annurca	nd	12.88 ± 0.89	0.259	0.42
Bacci	12.04 ± 1.24	3.24 ± 0.09	0.881	3.34
Pelingo	3.96 ± 0.23	1.81 ± 0.09	1.996	28.39

nd: not detectable

### The effect of apple juice on cell proliferation

**Fig 1:**
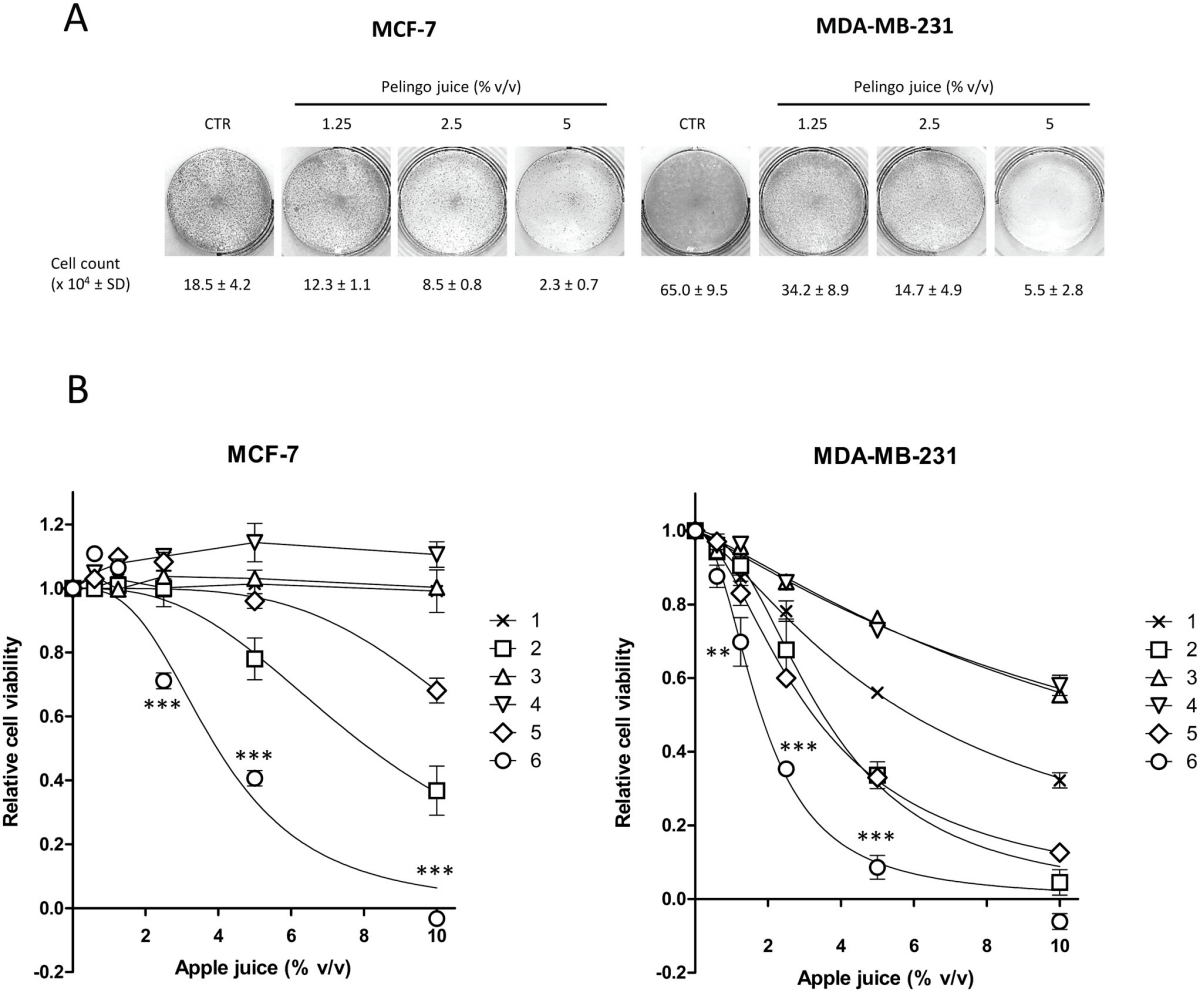
Effect of apple juice on cell viability in MCF-7 and MDA-MB-231 cell lines. (A) Anchorage-dependent cell growth assay. Cells (5x10^4^) were treated with Pelingo juice for 72 h. Cell growth was evaluated by cell count and crystal violet staining. (B) Cell viability assay. Cells (5x10^3^) were treated for 72 h with six apple juice cultivars: 1, Abbondanza white pulp. 2, Abbondanza red pulp. 3, Marchigiana pink pulp. 4, Annurca. 5, Bacci. 6, Pelingo. Cell viability was evaluated by MTS assay. Data are expressed as means ± SEM of at least three separate experiments. Asterisks indicate significantly differences of Pelingo activity with respect to the same dose of other apples (2-way ANOVA followed by Bonferroni post hoc test. ** *p*<0.01; *** *p*<0.001).

The antiproliferative activity of Pelingo juice was evaluated with anchorage-dependent growth inhibition assay. As shown in Fig 1A, Pelingo juice markedly inhibited cell growth from 2.5% v/v in MCF-7 (54.1% growth inhibition), and from 1.25% v/v in MDA-MB-231 (47.4% growth inhibition) cells.

The effect of Pelingo juice was then compared with five other apple juice cultivars using the MTS assay. Pelingo juice significantly reduced cell proliferation in a dose dependent manner in MCF-7 and MDA-MB-231 cells (1-way ANOVA followed by Bonferroni *post hoc* test, *p*<0.001), with IC_50_ of 3.96 ± 0.23 and 1.81 ± 0.09% v/v, respectively (Fig 1B). Moreover, Pelingo juice was found to be significantly the most active in both cell lines, followed by Abbondanza red pulp (2-way ANOVA followed by Bonferroni *post hoc* test, ** *p*<0.01, *** *p*<0.001) (Fig 1B and Table 1).

The correlation between the proliferative index and IC_50_ was also evaluated, using two additional cell lines, WI-38 and NIH-3T3. The results showed that antiproliferative activity was directly proportional to the rate of replication of the cells tested. In fact, the IC_50_ value was lower (higher activity) in cells with fast growth (MDA-MB-231 and NIH-3T3 cells; proliferative index: 5.45 and 4.23 respectively; IC_50_: 1.81 and 1.13% v/v respectively), than in cells with slow growth (MCF-7 and WI-38 cells; proliferative index: 0.48 and 0.63 respectively; IC_50_: 3.96 and 4.34% v/v respectively).

The activity of the Pelingo juice stored at -20°C was stable over a 1-year period (not shown). We also found that the activity of Pelingo juice obtained from peeled apple was not significantly different to that of whole apple (not shown).

### Analysis of cell cycle perturbations

**Fig 2:**
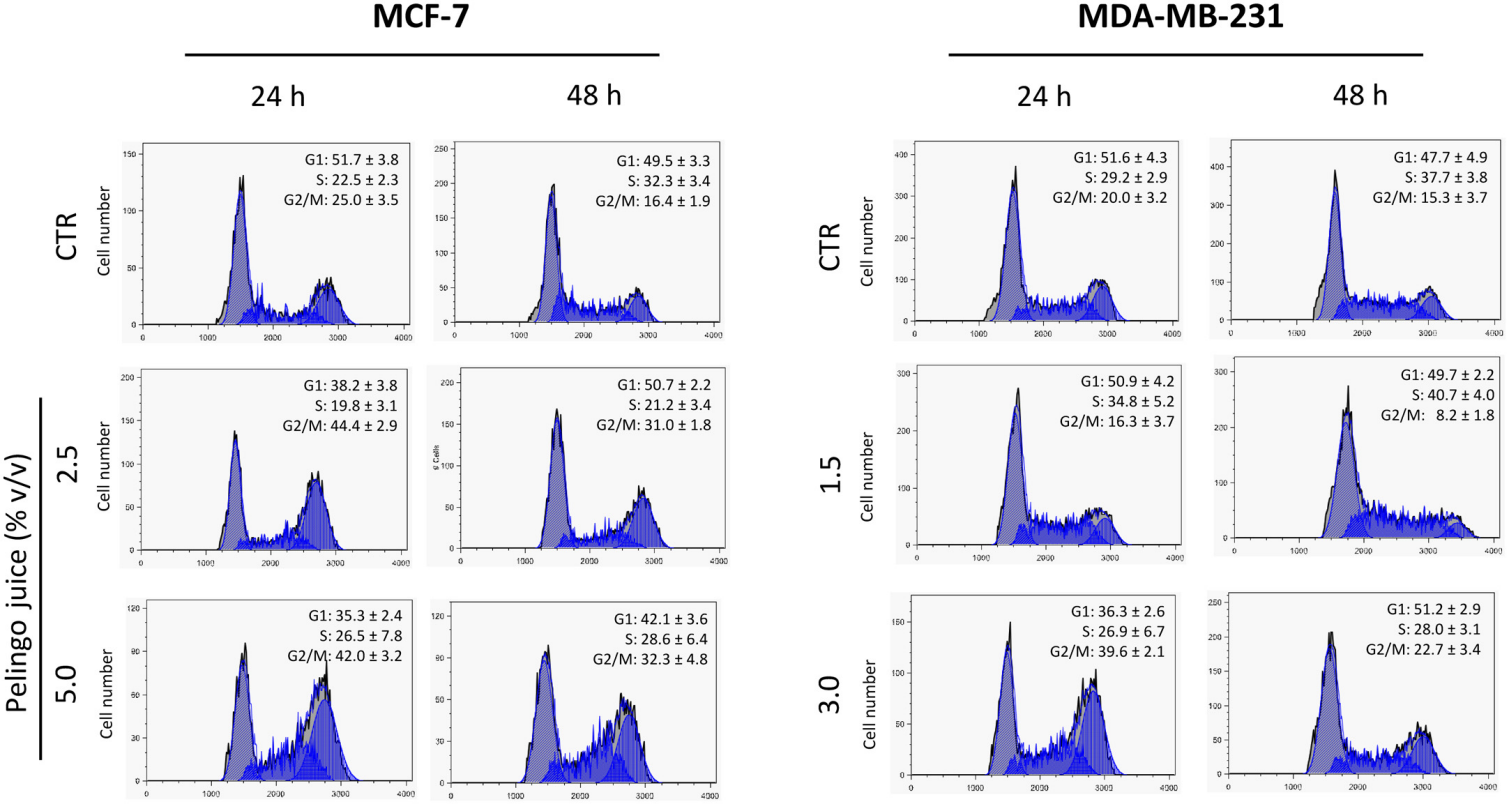
Cell cycle perturbation in MCF-7 and MDA-MB-231 cells treated with Pelingo juice. Cells were treated for 24 and 48 h with 2.5 and 5% v/v (MCF-7) and 1.5 and 3.0% v/v (MDA-MB-231) of Pelingo juice and stained with propidium iodide. DNA content profiles were analyzed by flow cytometry. Images are representative of one experiment of three replicates. Data are represented as means ± SD.

Cell cycle analysis was carried out in MCF-7 and MDA-MB-231 cells to evaluate the effect of Pelingo juice on cell cycle progression. Cells were treated with Pelingo juice for 24 and 48 hours at the final concentrations of 2.5 and 5.0% v/v for MCF-7 and 1.5 and 3.0% for MDA-MB-231 and then stained with propidium iodide for flow cytometric analysis. Results showed that Pelingo juice induced a dose-dependent cells accumulation in late S and G2/M phases of the cell cycle in both MCF-7 and MDA-MB-231 cell lines (Fig 2). In fact, after 24 hours of treatment with Pelingo juice, the cellular population in G2/M phase significantly increased from 25.0 ± 3.5% to 42.0 ± 3.2% in MCF-7 and from 20.0 ± 3.2% to 39.6 ± 2.1% in MDA-MB-231 (1-way ANOVA followed by Dunnett's multiple comparison test. *p*<0.001).

Moreover, results show that the Pelingo juice did not significantly induces sub-G1 events (S2 and S3 Figs), which would support the evidence for apoptosis. In MDA-MB-231, supra-G2 events were revealed (S3 Fig), which may suggest evasion of mitotic arrest and need to be further investigated.

### The effect of Pelingo juice on morphological features

Induction of apoptosis/necrosis/autophagy was simultaneously assessed by colorimetric analyses using Hoechst, propidium iodide and acridine orange dyes. MCF-7 and MDA-MB-231 cells were treated with 2.5 and 5% v/v Pelingo juice for 24, 48 and 72 h, stained as described in the methods section and observed under a fluorescence microscope. Propidium iodide positive nuclei (necrotic cells) show necrosis induction after 24 and 48 h of Pelingo juice treatment in MCF-7 and MDA-MB-231 cells, respectively (S4 Fig) without morphological features typical of apoptosis, such as nuclear fragmentation, confirming the results from cytofluorimetric analyses. Moreover, in both cell lines we can observe an induction of vacuolization and the presence of acidic vesicular organelles (AVOs) following juice treatment, that could be indicate a possible autophagy induction [36].

### Analysis of ERK1/2 activity, p21 levels and LC3B-II/LC3B-I ratio in breast cancer cells

**Fig 3:**
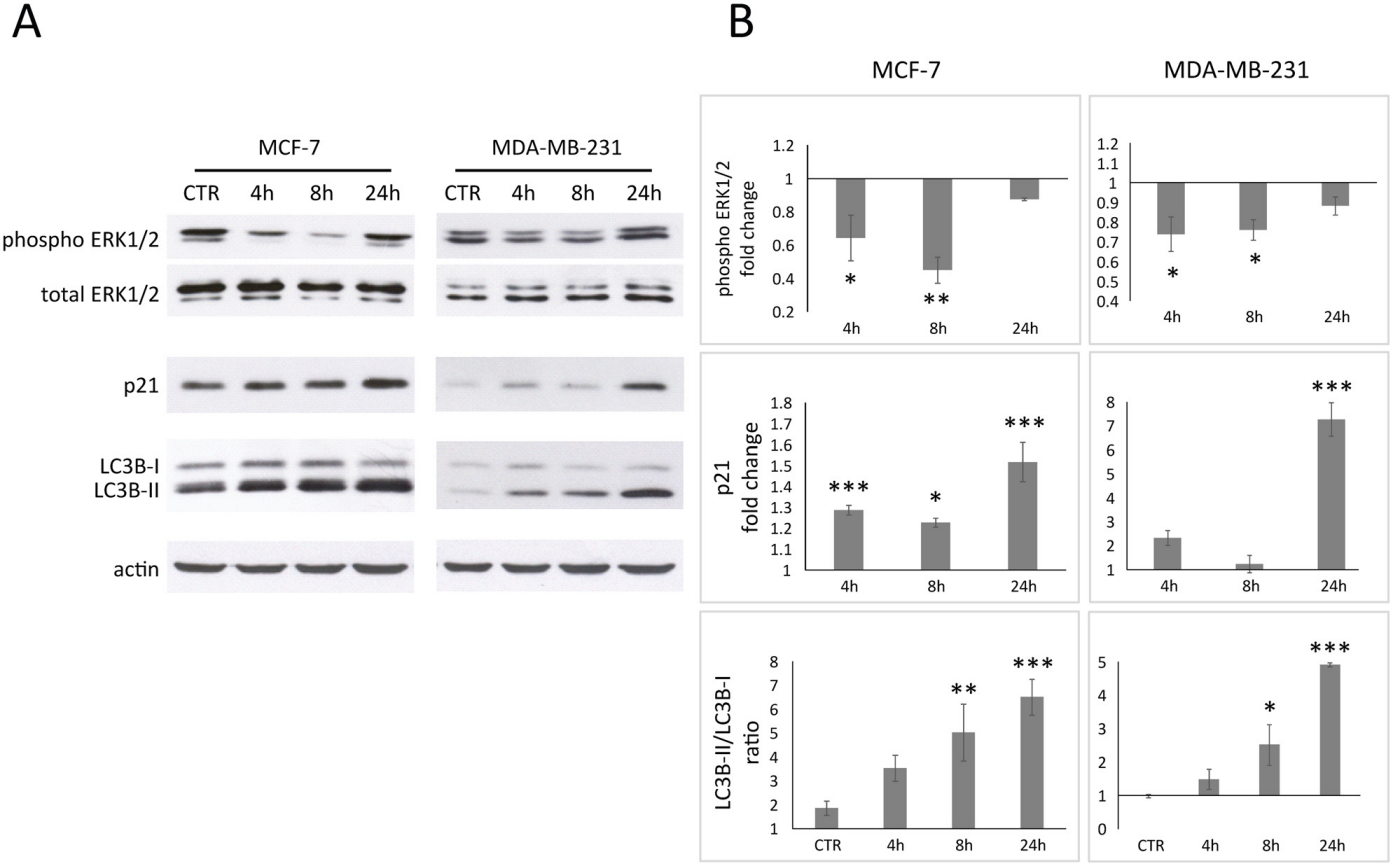
Western blot analysis of MCF-7 and MDA-MB-231 cells treated for 4, 8 and 24 h with 2.5% v/v of Pelingo juice. ERK1/2 phosphorylation, p21 overexpression and LC3B-II/LC3B-I ratio were analyzed in total cell extracts. (A) Representative images of three experiments giving similar results. (B) Densitometric analysis: p21 and phospho-ERK1/2 are shown as fold changes relative to control; changes in autophagosomes-related protein LC3B are shown as LC3B-II(lipidated)/LC3B-I(non lipidated) ratio. Phospho-ERK was normalized to total ERK; p21, LC3B-II and LC3B-I were normalized to actin. Data are expressed as means ± SEM of three replicates. Asterisks indicate significantly differences respect to control (1-way ANOVA followed by Dunnett's multiple comparison test. * *p*<0.05; ** *p*<0.01; *** *p*<0.001).

To further investigate the cytostatic activity of Pelingo juice revealed in cytofluorimetric analysis, the level of p21 and activity of ERK1/2 were evaluated. Both proteins are involved in G2 cell cycle arrest and cell proliferation. Results shows that p21 was time-dependent upregulated with about 1.5-fold and 7.0-fold in MCF-7 and MDA-MB-231 respectively after 24 hours of treatment (Fig 3) (*p*<0.01). The activity of ERK1/2 was evaluated using a phospho-sensitive antibody, revealing an inhibition of ERK1/2 activity in both MCF-7 and MDA-MB-231. The maximum effect was after 8 hours of treatment with about 0.4-fold and 0.7-fold to total-ERK in MCF-7 and MDA-MB-231, respectively (Fig 3) (*p*<0.05). Autophagic process could be monitored evaluating the accumulation of LC3B conjugated to phosphatidylethanolamine (the autophagosome membrane-bound form, LC3B-II) respect to the non-lipidated form (LC3B-I) [36]. Results show an increase of LC3B-II/LC3B-I ratio from 1.8 and 1 in untreated cells to 6.5 and 5.0 in MCF-7 and MDA-MB-231 treated for 24 h, respectively (*p*<0.001), confirming the results obtained with acridine orange staining.

### Inhibition of *in vitro* tumorigenesis of MDA-MB-231 cells by Pelingo juice

**Fig 4:**
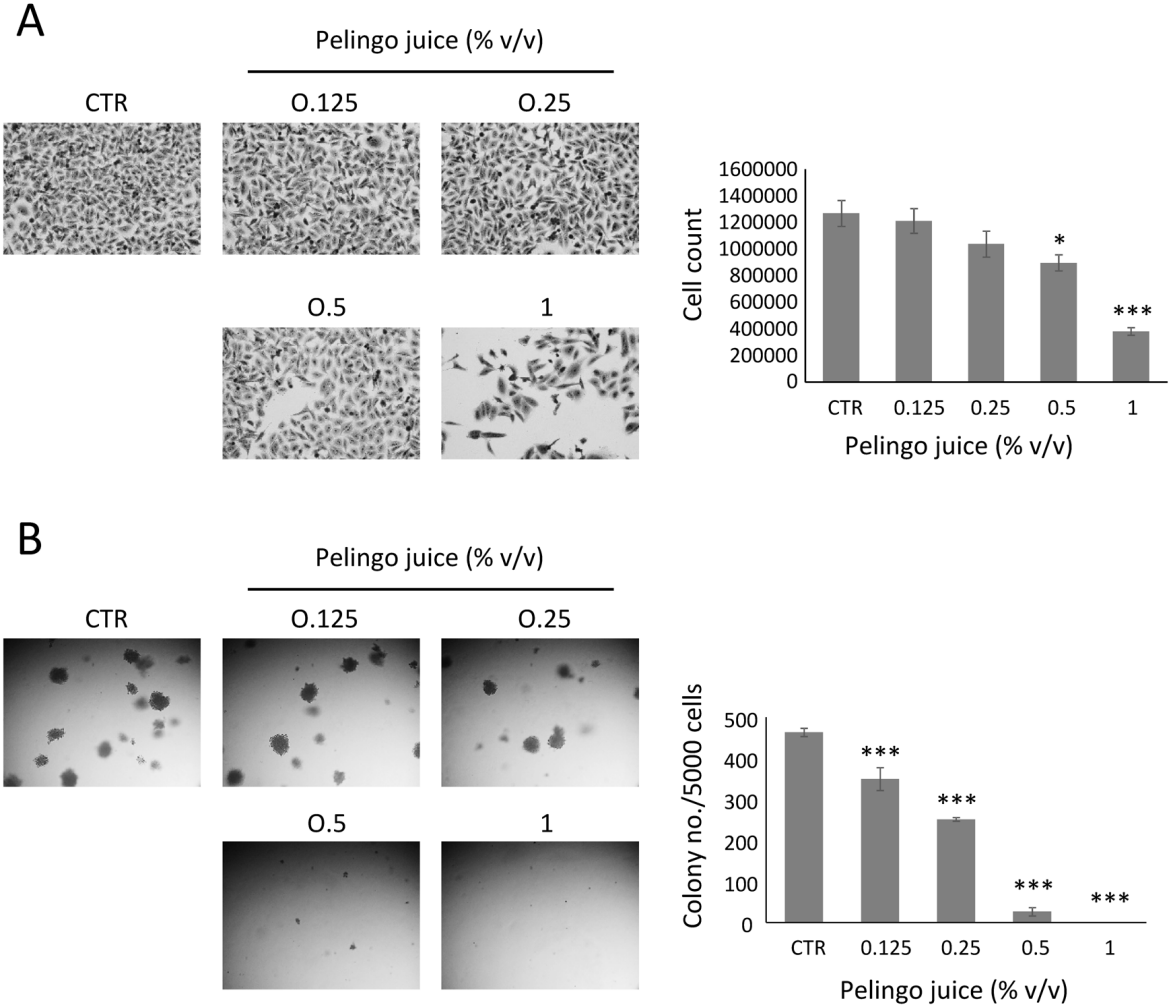
Pelingo juice inhibits *in vitro* tumorigenesis of MDA-MB-231 cells. (A) Anchorage-dependent cell growth assay. Cells (5x10^4^) were treated for 72 h with 0.125, 0.25, 0.5 and 1% v/v of Pelingo juice. Cell growth was evaluated by cell count and crystal violet staining. (B) Anchorage-independent cell growth assay. Cells (5x10^3^) were growth in soft agar with 0.125, 0.25, 0.5 and 1% v/v of Pelingo juice for 14 days and colonies containing more than 20 cells were counted. Data are expressed as means ± SEM of three separate experiments. Asterisks indicate significantly differences respect to control (1-way ANOVA followed by Dunnett's multiple comparison test. * *p*<0.05; *** *p*<0.001).

The effect of Pelingo juice at non-cytotoxic doses (0.125, 0.25, 0.5 and 1% v/v) on MDA-MB-231 *in vitro* tumorigenesis was examined in both anchorage-dependent and anchorage-independent growth assays. Results of anchorage-dependent growth assay showed that Pelingo juice did not inhibit cell viability up to 1% v/v, whereas showed a significant cytostatic effect at 0.5 and 1% v/v (Fig 4A). The MDA-MB-231 cells were then cultured in soft agar with increasing concentrations of Pelingo juice (0.125, 0.25, 0.5 and 1% v/v) for 14 days to evaluate the effect on anchorage-independent cell growth. Results in Fig 4B showed that Pelingo juice significantly suppressed the colony formation at 0.125, 0.25 and 0.5% v/v, showing a complete inhibition at 1% v/v (*p*<0.001).

### Inhibition of TPA-induced JB6 cell transformation by Pelingo juice

**Fig 5:**
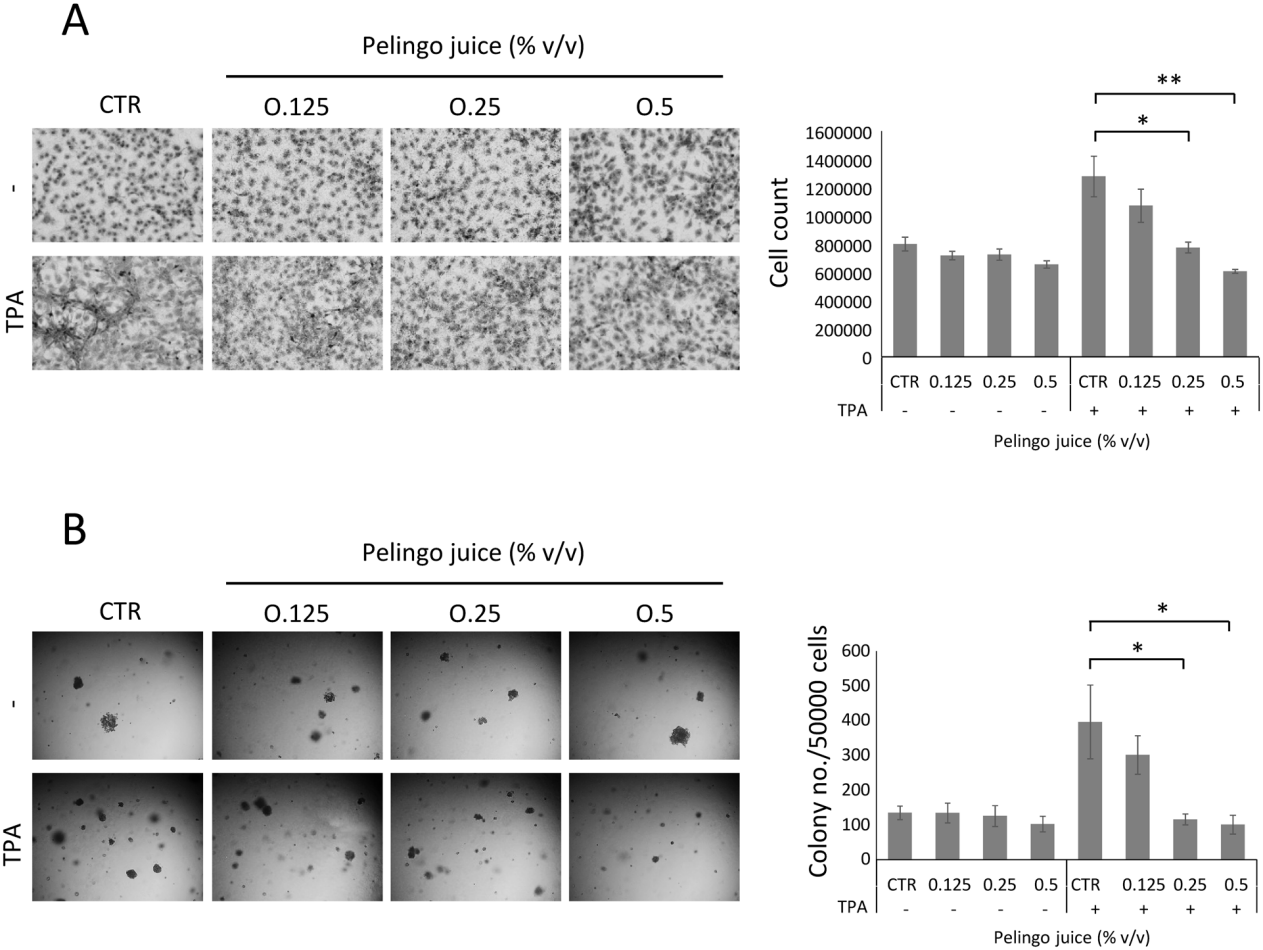
Pelingo juice inhibits TPA-induced transformation of JB6 P+ cells. (A) Anchorage-dependent cell growth assay. Confluence cells (3x10^5^) were stimulated with 10 ng/ml of TPA and treated with 0.125, 0.25 and 0.5% v/v of Pelingo juice for 72 h. Cell growth was evaluated by cell count and crystal violet staining. (B) Anchorage-independent cell growth assay. Cells (5x10^4^) were growth in soft agar with 10 ng/ml of TPA and 0.125, 0.25 and 0.5% v/v of Pelingo juice for 21 days and colonies containing more than 20 cells were counted. Data are expressed as means ± SEM of three separate experiments. Asterisks indicate significantly differences respect to control (1-way ANOVA followed by Dunnett's multiple comparison test. * *p*<0.05; ** *p*<0.01).

The effect of Pelingo juice treatment on the TPA-induced anchorage-dependent and anchorage-independent cell growth was evaluated in JB6 P+ cells (Fig 5). To evaluate the effect on anchorage-dependent cell growth, cells were cultured at confluence and simultaneously stimulated with 10 ng/ml TPA and treated with Pelingo juice. Results in Fig 5A showed that Pelingo juice did not inhibit cell viability of JB6 P+ cells up to 0.5% v/v, whereas significantly inhibited TPA-induced anchorage-dependent cell growth at 0.25 and 0.5% v/v (*p*<0.05 and *p*<0.01 respectively).

The effect on anchorage-independent cell growth was evaluated in soft-agar, monitoring the TPA-induced colony formation of JB6 cells (Fig 5B). Cells were treated with increasing concentration of Pelingo juice, stimulated with 10 ng/ml of TPA and cultured in soft agar for 21 days. Results showed that Pelingo juice completely suppressed the TPA-induced colony formation at 0.25 and 0.5% v/v (*p*<0.05) (Fig 5B). These results indicate that Pelingo juice could has a chemopreventive potential against TPA-induced tumorigenesis in JB6 P+ cells.

### Analysis of ERK1/2 activity in JB6 P+ cells

**Fig 6:**
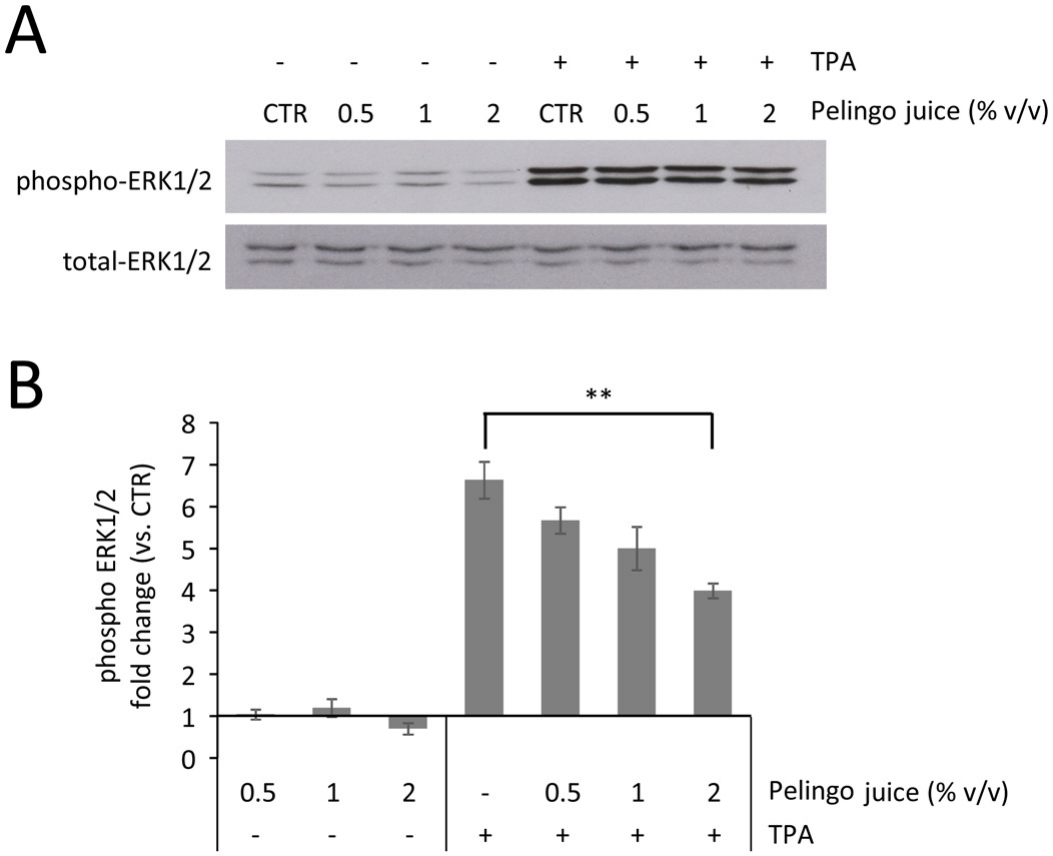
Western blot analysis of JB6 P+ cells treated with Pelingo juice and TPA. Cells were pre-treated with 0.5, 1 and 2% v/v of Pelingo juice for 24 h and stimulated with 10 ng/ml of TPA. ERK1/2 phosphorylation was analyzed in total cell extracts. (A) Representative images of three experiments giving similar results. (B) Densitometric analysis of phospho-ERK1/2 respect to control. Phospho-ERK was normalized to total ERK. Data are expressed as means ± SEM of three replicates. Asterisks indicate significantly differences respect to control (1-way ANOVA followed by Dunnett's multiple comparison test. ** *p*<0.01).

The effect of Pelingo juice on TPA-induced tumorigenesis was further evaluated analyzing the activation of ERK1/2, kinase involved in the neoplastic transformation induced by TPA. JB6 P+ cells were pre-treated with Pelingo juice for 24 h and stimulated with 10 ng/ml of TPA for 60 min. The phosphorylation of ERK1/2 was analyzed in total cell lysates, revealing that Pelingo juice partially inhibited the TPA-induced ERK1/2 phosphorylation at 2% v/v (*p*<0.01) (Fig 6).

## Discussion

During the past decade, breast cancer has become the most common form of cancer among women in Western countries, and the number of cases diagnosed worldwide is increasing [1]. Surgical interventions and chemotherapy are able to significantly reduce mortality; however, both of these interventions have several limitations such as reduced efficacy in the metastatic phase, potential toxicity and resistance to antiestrogen compounds. Thus, new approaches to combat this neoplasm are needed, also taking into account the fact that it is far easier to prevent cancer that to cure the disease. In this contest, in recent years the development of a new approach to cancer prevention, called chemoprevention, has aroused growing interest. Chemoprevention is defined as the use of agents of natural or synthetic origin to try to impede, arrest, or reverse carcinogenesis before a complex series events leads to invasive and metastatic malignancy [37, 38]. Prevention of cancer through dietary intervention has become an important issue. Concerning natural agents, epidemiological studies have documented links between intake of fruits and vegetables and reduced risk of breast cancer [2] and *in vitro* studies have found antiproliferative properties of phytochemicals derived from natural compounds [39, 40]. It is now widely believed that the health benefits of fruits and vegetables are likely due to the additive and synergistic effects of a range of phytochemicals, and such benefits cannot be provided through a dietary supplements alone [1, 21, 41].

In this study, have shown that whole juice from a new cultivar of apple with reddish pulp, called the Pelingo apple, is a potent growth inhibitor of human breast cancer cells *in vitro*. Consistent with that which was reported by Liu *et al*. [42], we have also demonstrated that different cultivars of apples have different antiproliferative activity on breast cancer cell lines. The high level of activity shown by Pelingo apple compared with other apple cultivars tested could be due to the exceptionally large amounts of bioactive compounds that these apple contain. Indeed, the chemical analysis performed in this investigation revealed a total polyphenol content 2–8 fold higher in the Pelingo apple than in the other cultivars tested. Moreover, total anthocyanin content was also evaluated, showing that the Abbondanza red pulp cultivar was the richest in anthocyanins, followed by the Pelingo apple. Anthocyanins are a class of compounds that confer to plants a typical blue-red color. In fact they are found in higher levels in those apple cultivars which show a more intense color, and it has been suggested that these compounds possess chemopreventive properties [43]. However, the lower activity of the Abbondanza red pulp apple compared to the Pelingo apple suggests that anthocyanins may be less involved in the antiproliferative activity of these cultivars [44].

Among the isolated polyphenolic compounds, the main flavonoids in apple are quercetin 3-*O*-*β*-D-glucopyranoside and 3-*O*-*β*-D-galactopyranoside, followed by minor amounts of quercetin, (-)-catechin, (-)-epicatechin, and quercetin 3-O-R-L-arabinofuranoside; quercetin and quercetin 3-O-β-D-glucopyranoside exhibited potent antiproliferative activities against MCF-7 cells [45]. In a previous work [30] the total polyphenolic content of Pelingo apple was detected and the chlorogenic acid, quercetin-3-O-galactoside, quercetin-3-O-glycoside, quercetin-3-O-pentoside, epicatechin, catechin were the main compounds. To the our knowledge, the high levels of these polyphenols were not found in other apple cultivars including Annurca and Red Delicious cultivar [46]. In particular, according to literature [47], we can assume that the higher levels of catechin, epicatechin and chlorogenic acid in Pelingo apple can determine the exhibited effects on tumorigenesis and on breast cancer cell proliferation.

In agreement with that which was reported by Sun *et al*. [22] regarding apples of the Red Delicious cultivar, the antiproliferative activity of the Pelingo apple was found to be more marked against the triple-negative MDA-MB-231 cells as compared to the ER+ MCF-7 cells. However, while Sun J *et al*. reported that the inhibition of cell proliferation occurs through a mechanism that induces G1 arrest [22], our data showed that the Pelingo apple induced cell accumulation in the G2/M phase of the cell cycle.

The cytostatic effect of Pelingo juice it was confirmed by the inhibition of ERK1/2 activity and the upregulation of p21. ERK1 and ERK2 participate in the Ras-Raf-MEK-ERK signal transduction cascade, implicated in the regulation of a large variety of processes including cell adhesion, cell migration, cell cycle progression and proliferation [48], including the G2/M transition [49]. The p21 protein is a universal inhibitor of the cyclin-dependent kinase (CDK) family and its upregulation is associated with cell cycle arrest in the G2/M phase [50]. The CDKs inhibitors could be regulated by different pathways such as PI3K and downstream AKT/protein kinase B family members, that may be implicated in both p53-dependent and p53-independent expression of p21/CDKN1A [51, 52]. Furthermore, our data revealed that the higher activity of juice on MDA-MB-231 cells is due to their higher replicative rate compared to MCF-7 cells, suggesting a biological effect related to the replication rate of the cellular population tested.

Although the induction of apoptosis in cancer cells has been previously shown using total apple extract [53], our results showed cell necrosis at the highest juice doses without any evidence of apoptosis induction.

On the contrary, the AVOs’ formation and lipidated LC3B accumulation indicate autophagy induction. This interesting mechanism has recently been proposed as a strategy for cancer prevention because it could be responsible for eliminating damaged proteins, organelles and DNA, all of which can contribute to mutation and initiate transformation in pre-malignant cells [54]. Pelingo juice also induces cellular vacuolization in both the cell lines tested. This morphologic modification has been observed in previous studies, in which the authors showed an induction of vacuolization in cells treated with prenylated flavones [55]. The induction of cellular vacuolization induced by the Pelingo apple could be due to the presence of the polyphenols observed in our analysis, and subsequent studies will focus on the identification of these compounds.

In this study, we evaluated the effect of Pelingo juice on TPA-induced tumorigenesis of the pre-neoplastic JB6 P+ cells. We found that Pelingo juice is able to block the TPA-induced neoplastic transformation, resulting in a inhibition of colony formation in semi-solid medium. Moreover, since TPA induces neoplastic transformation through activation of the ERKs pathway in various cells [56], we demonstrated that Pelingo juice partially inhibited the TPA-induced ERK1/2 activity. These results suggest that Pelingo apple could have a chemopreventive potential against TPA-induced tumorigenesis in JB6 P+ cells. Interestingly, in the previous study by Giomaro *et al*. [30], chemopreventive compounds such as catechins and procyanidins have been identified [13].

The approach to developing new chemopreventive agents has changed considerably in recent years and now involves the extensive preclinical mechanistic study of agents, such as inhibition of proliferation, induction of autophagy or apoptosis and differentiation, before clinical trials are initiated [37, 57].

## Conclusion

In conclusion, we have shown that Pelingo apple is characterized by a very high polyphenol content which strongly inhibits the proliferation of breast cancer cells, induces cell accumulation in the G2/M phase of the cell cycle and autophagy, overexpression of p21 and inhibition of ERK1/2 activity. The Pelingo apple also suppress TPA-induced *in vitro* neoplastic transformation through inhibition of ERK1/2 activity. This findings suggest that Pelingo apple could provide bioactive non-nutrient compounds with potential chemopreventive activity.

## Supporting Information

S1 Fig A representative image of the Pelingo apple. (TIF)

S2 Fig Cell cycle effects of Pelingo juice in MCF-7 cells. DNA content profiles of cells exposed for 24 and 48 h to 2.5 and 5.0% v/v, stained with propidium iodide, and analyzed by flow cytometry are shown as ungated cellular events and in a logaritmic scale.
(TIF)

S3 Fig Cell cycle effects of Pelingo juice in MDA-MB-231 cells. DNA content profiles of cells exposed for 24 and 48 h to 1.5 and 3.0% v/v, stained with propidium iodide, and analyzed by flow cytometry are shown as ungated cellular events and in a logaritmic scale.
(TIF)

S4 Fig Evaluation of apoptotic/necrotic/autophagic processes in MCF-7 (left panel) and MDA-MB-231 (right panel). Cells were treated with 2.5 and 5.0% v/v of Pelingo juice for 24, 48 and 72 h, and directly stained with Hoechst, propidium iodide and acridine orange. Blue excitation filter was used for acridine orange; the cytoplasm and nucleus fluoresce green, whereas acidic compartments fluoresce orange-red. UV excitation was used for Hoechst and propidium iodide; undamaged cells nuclei fluoresce blue, necrotic cells nuclei fluoresce red.
(TIF)
