# Supplementary figures and images for: Inhibition of Breast Cancer Cell Proliferation and In Vitro Tumorigenesis by a New Red Apple Cultivar
Source: PLoS One. 2015 Aug 18;10(8):e0135840. doi: 10.1371/journal.pone.0135840 (PMC4540469; doi:10.1371/journal.pone.0135840)

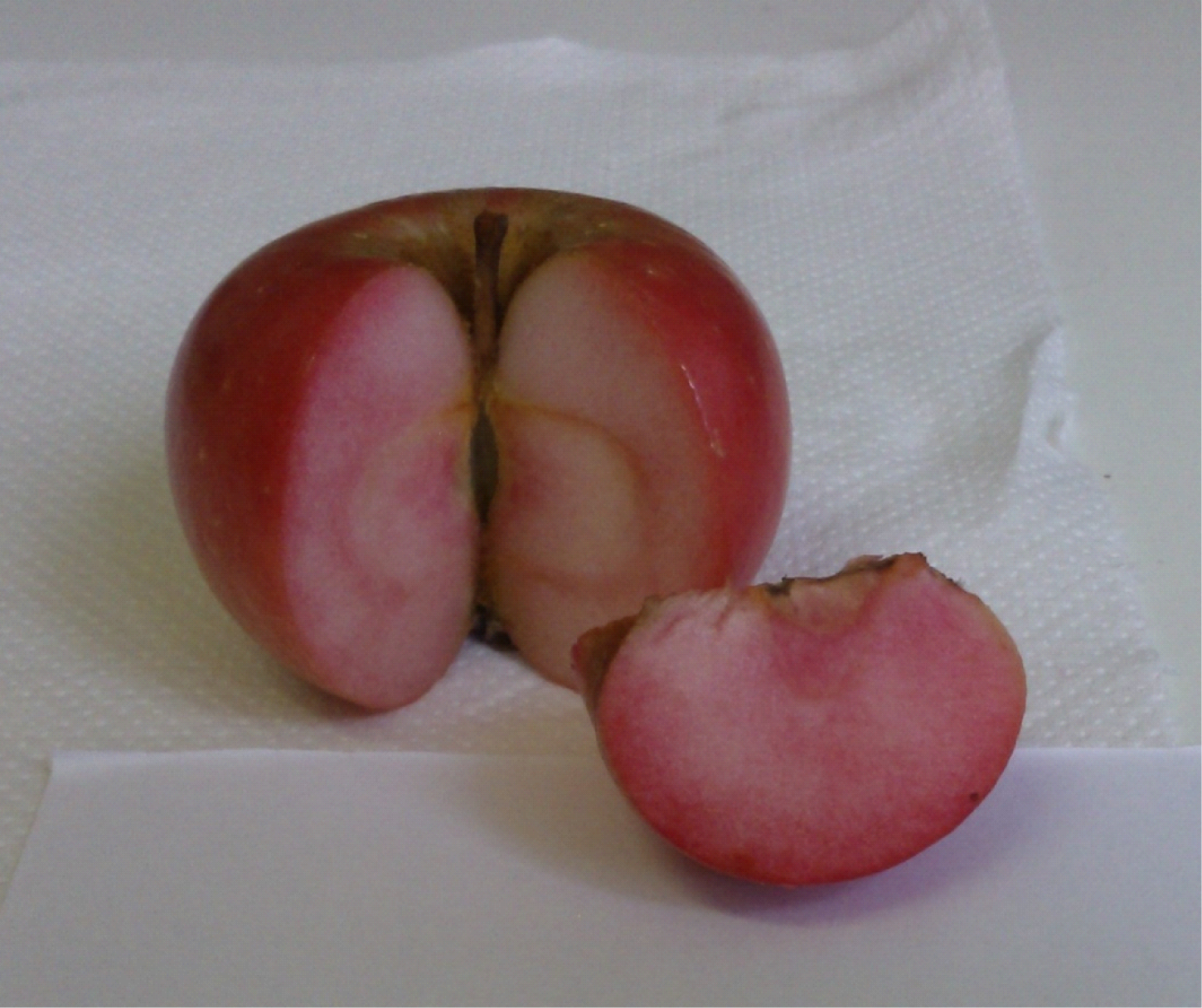

Supplement: S1 Fig — (TIF) [file pone.0135840.s001.tif]

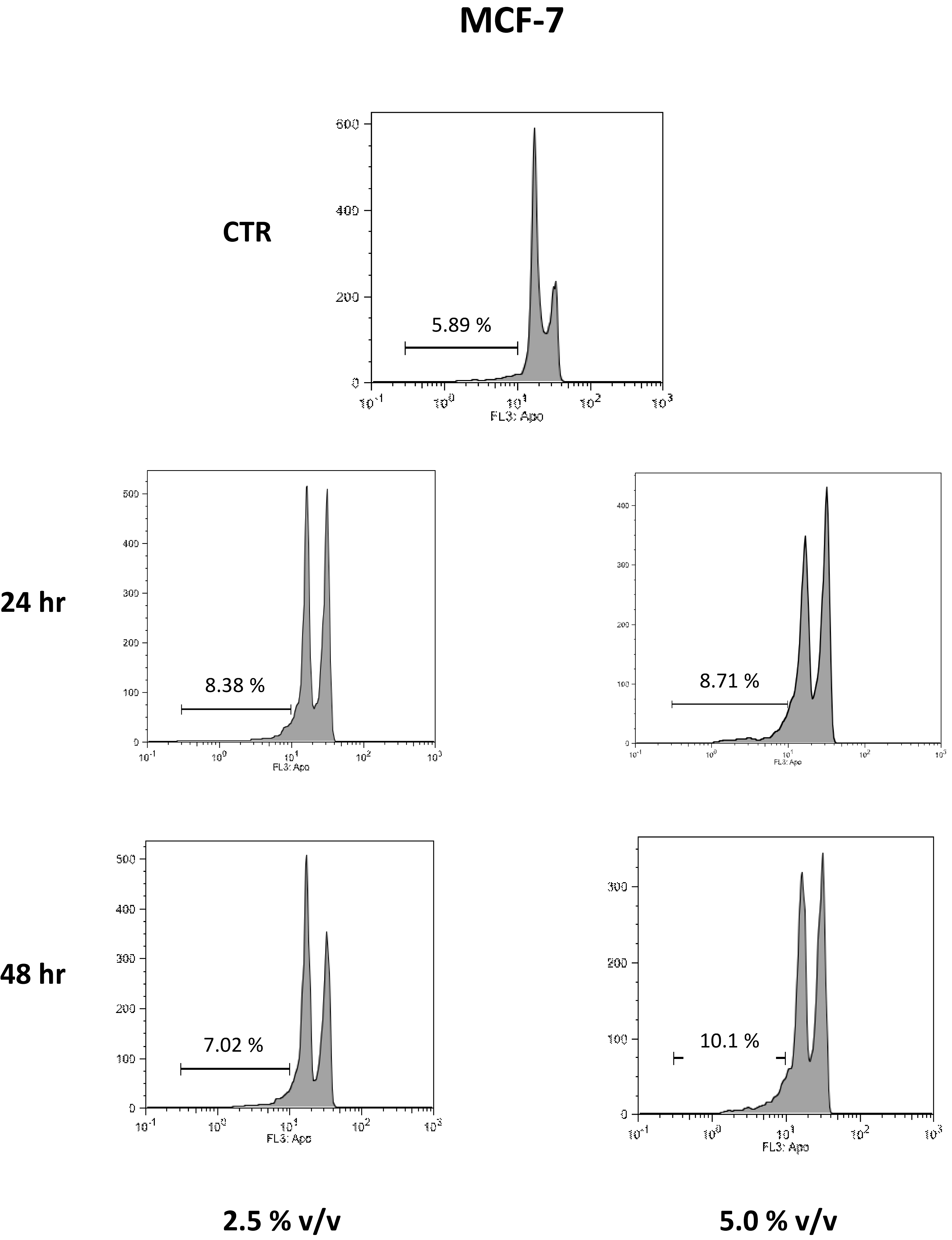

Supplement: S2 Fig — DNA content profiles of cells exposed for 24 and 48 h to 2.5 and 5.0% v/v, stained with propidium iodide, and analyzed by flow cytometry are shown as ungated cellular events and in a logaritmic scale. (TIF) [file pone.0135840.s002.tif]

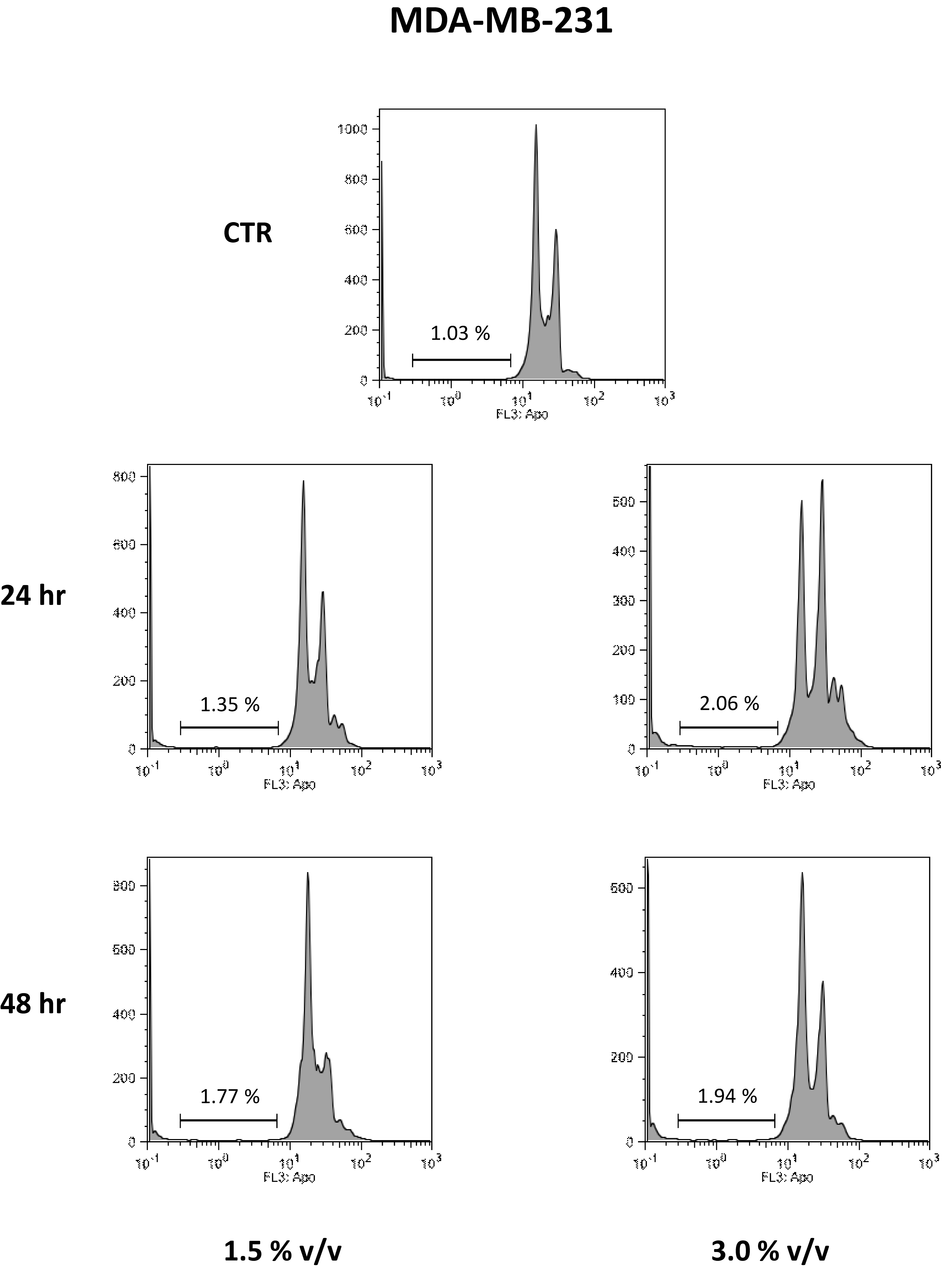

Supplement: S3 Fig — DNA content profiles of cells exposed for 24 and 48 h to 1.5 and 3.0% v/v, stained with propidium iodide, and analyzed by flow cytometry are shown as ungated cellular events and in a logaritmic scale. (TIF) [file pone.0135840.s003.tif]

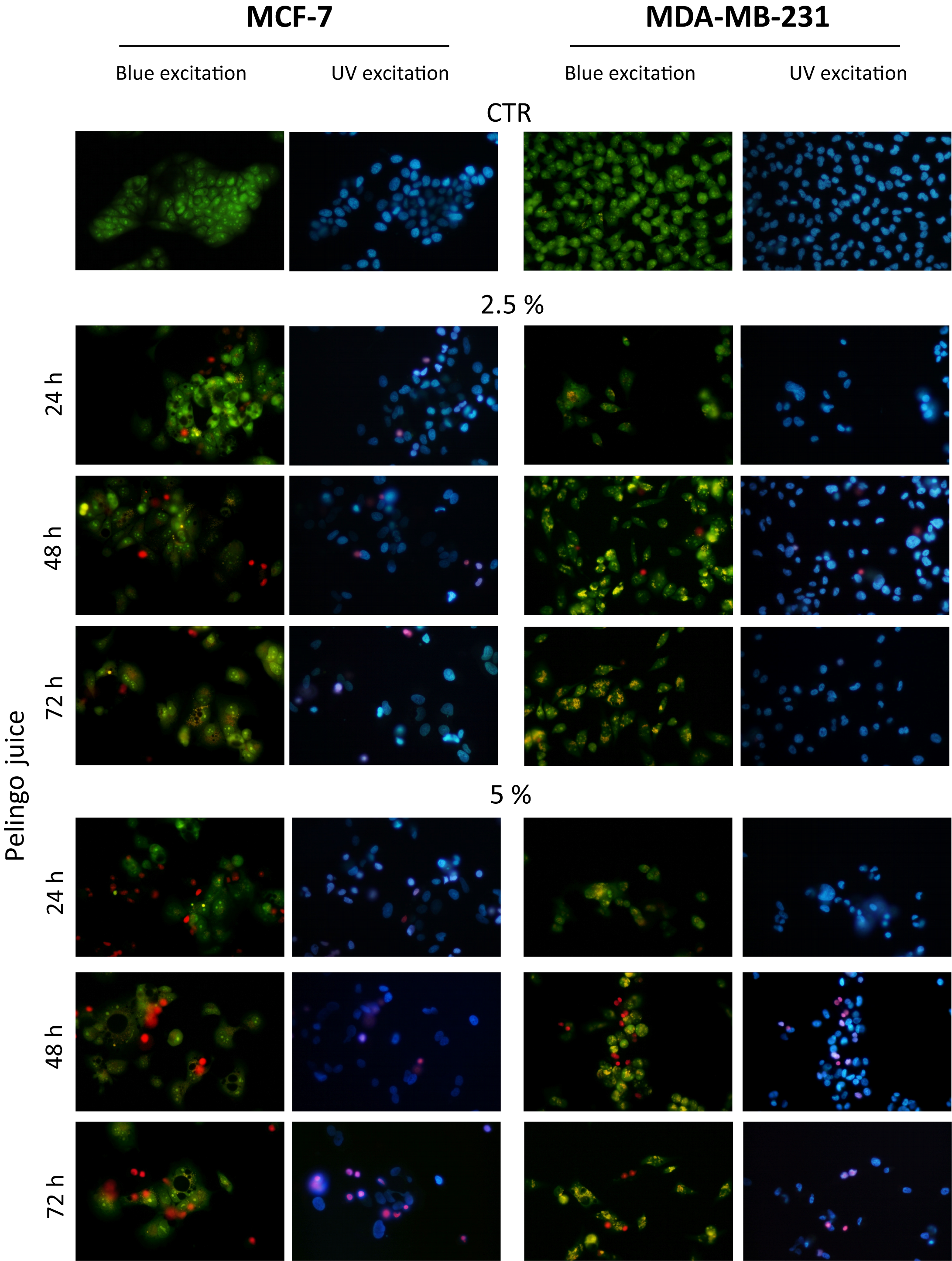

Supplement: S4 Fig — Cells were treated with 2.5 and 5.0% v/v of Pelingo juice for 24, 48 and 72 h, and directly stained with Hoechst, propidium iodide and acridine orange. Blue excitation filter was used for acridine orange; the cytoplasm and nucleus fluoresce green, whereas acidic compartments fluoresce orange-red. UV excitation was used for Hoechst and propidium iodide; undamaged cells nuclei fluoresce blue, necrotic cells nuclei fluoresce red. (TIF) [file pone.0135840.s004.tif]
